# Clinical significance of serum magnesium levels in patients with heart failure with preserved ejection fraction

**DOI:** 10.1097/MD.0000000000017069

**Published:** 2019-09-20

**Authors:** Taiki Nishihara, Eiichiro Yamamoto, Daisuke Sueta, Koichiro Fujisue, Hiroki Usuku, Fumi Oike, Masafumi Takae, Yuichiro Arima, Satoshi Araki, Seiji Takashio, Taishi Nakamura, Satoru Suzuki, Kenji Sakamoto, Hirofumi Soejima, Hiroaki Kawano, Koichi Kaikita, Kenichi Tsujita

**Affiliations:** Department of Cardiovascular Medicine, Faculty of Life Sciences, Graduate School of Medical Science and Center for Metabolic Regulation of Healthy Aging (CMHA), Kumamoto University, Kumamoto, Japan.

**Keywords:** heart failure, HFpEF, magnesium

## Abstract

Although serum magnesium (Mg) levels are closely associated with the prognosis of heart failure (HF) patients, the clinical significance of sMg levels on the cardiovascular outcomes of HF with preserved ejection fraction (HFpEF) patients is not fully understood. This study was a retrospective, single-center, observational study. We enrolled 452 consecutive HFpEF patients admitted to Kumamoto University Hospital. We defined lower sMg as <2.0 mg/dl (=0.8 mmol/L) based on recent clinical evidence and compared their clinical characteristics and prognosis. There were no significant differences between groups in the use of all medications (loop diuretics, mineralocorticoid receptor antagonists, renin-angiotensin-aldosterone system inhibitors, calcium channel blockers, beta blockers, statins, and Mg preparations). The lower sMg group showed a significantly higher prevalence of diabetes mellitus (DM), uric acid levels, and BNP levels compared with the higher sMg group. Kaplan–Meier curve revealed a significantly higher probability of HF-related events in the lower sMg group compared with the higher sMg group (log-rank test, *P* = .012). Multivariate Cox-proportional-hazard analysis revealed that the lower sMg group had significantly and independently higher probabilities of HF-related events compared with the higher sMg group (hazard ratio = 2.37, 95% confidence intervals = 1.27–4.41, *P* = .007). We reclassified the risk of HF-related events after adding the lower sMg to the other prognostic factors (age, previous hospitalization for HF, DM, Ln-BNP); the continuous net reclassification improvement was 29.0% (*P* = .041). sMg levels might provide important prognostic information in regard to HFpEF.

## Abstract 2

Supplemental Digital Content is available in the text

## 1 Introduction

In heart failure (HF) patients, various factors, such as hyperactivity of the renin-angiotensin system (RAS), influence of drug therapy (loop and thiazide diuretics), undernutrition,^[1,2]^ and others, can causes hypokalemia and hypomagnesemia. These conditions are well known to increase the risk of arrhythmia and sudden death. When diuretics in the treatment of HF, hypomagnesemia can lead to complications, which complicates arrhythmia and causes refractory hypokalemia; thus, the serum magnesium (sMg) concentration levels of HF patients should be determined. Hypomagnesemia has been reported to be an independent risk factor for cardiovascular disease,^[3–5]^ and replacement therapy is considered necessary in terms of long-term prognosis. The usefulness of Mg replacement therapy has been examined in several large-scale clinical studies targeting myocardial infarction, but until recently, their results were not consistent.^[6–8]^

Accumulating clinical studies have demonstrated that HF with reduced left ventricular (LV) ejection fraction (EF) (HFrEF) and HF with preserved LV ejection LVEF (HFpEF) are separate pathological conditions because of differences in survival rates^[9,10]^ and effective drug therapies. We have already reported that the blood sodium concentration,^[11]^ blood potassium concentration,^[12]^ plasma neopterin concentration,^[13]^ pulse pressure,^[14]^ and H_2_FPEF score^[15]^ are potent prognostic factors. Despite numerous papers have reported the importance of Mg deficiency in HF,^[16,17]^ few references have reported optimal sMg values in HFpEF patients. In this article, our aim was to determine the importance of monitoring sMg levels for HFpEF. The main purpose of this study was to investigate the relationship between the occurrence of future HF-related events and sMg levels in HFpEF patients.

## 2 Method

### 2.1 Ethics statement

All procedures were conducted in accordance with the Declaration of Helsinki and its amendments. Detailed ethics statement is available in the Supplemental Content.

### 2.2 Study subjects

**Figure 1:**
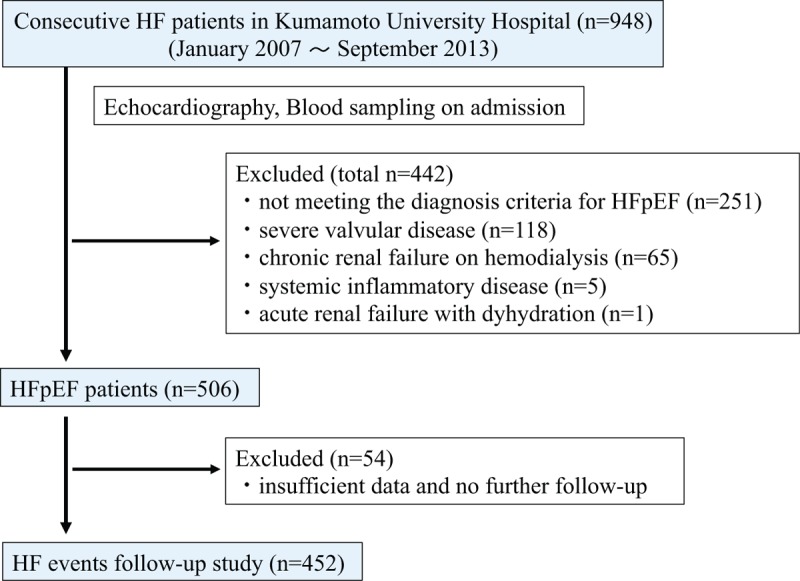
Flow chart showing the enrolment protocol. HF = heart failure, HFpEF = HF with preserved left ventricular ejection fraction.

We retrospectively investigated 948 consecutive patients with HF who were hospitalized in the Kumamoto University Hospital between January 2007 and September 2013 and recorded each patient's medical history and relevant clinical characteristics. We excluded 442 patients for the following reasons: severe valvular disease (n = 118), chronic renal failure, and undergoing hemodialysis (n = 65), systemic inflammatory disease (n = 5), acute renal failure with dehydration (n = 1). Because these diseases were known to have poor prognosis. And we excluded patients who were failure to meet the diagnostic criteria for HFpEF as described below (including HFrEF) (n = 253). Finally, the remaining 452 HFpEF patients, excluding those with insufficient data, were enrolled in this study (Fig. 1).

### 2.3 Clinical parameters and echocardiography

Detailed clinical parameters and echocardiography are available in the Supplemental Content.

### 2.4 Biochemistry

Both compensated and acute decompensated HF patients were registered in the present study. Venous samples were obtained in the early morning while the patient was in a stable and fasting state to measure sMg and other biochemical markers levels on admission. We defined lower sMg as <2.0 mg/dl (=0.8 mmol/L) based on recent review concerning the relationship between sMg levels and cardiovascular events.^[18]^ Detailed other blood sampling methods are available in the Supplemental Content.

### 2.5 Definition and severity of HFpEF

HFpEF was clinically defined according to the European Society of Cardiology task force as follows:

1. symptoms or signs of HF;
2. normal or mildly reduced LVEF (LVEF >50% and LV end-diastolic volume index <97 ml/m^2^); and
3. evidence of abnormal LV relaxation, filling, diastolic distensibility, and diastolic stiffness.

We excluded HFpEF patients who had shown even a transient reduction in ejection fraction. Hence, HFpEF patients whose LVEF was <50% and was improved by optimal medical therapy were not included in the present study. In our study, we stratified patients by the E/e′ ratio, grouped by either a ≥15 ratio or >8 but <15 ratio, and by BNP levels, with a cut-off of 100 pg/ml. Physicians further confirmed patients had HF by determining the New York Heart Association (NYHA) functional class,^[19]^ which was assessed under stable conditions after optimal therapy by the standard questionnaire.

### 2.6 Follow-up and HF-related events

Detailed follow-up and definitions of HF-related enents are available in the Supplemental Content.

### 2.7 Statistical analysis

Detailed statistical analysis is available in the Supplemental Content.

## 3 Results

### 3.1 Subjects

**Figure 2:**
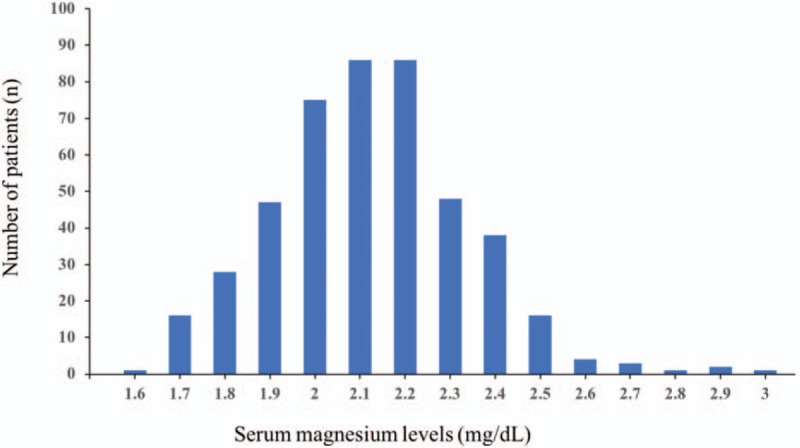
Distribution of serum magnesium levels in patients with HFpEF.

A total of 452 patients with HFpEF were enrolled in this study. The distribution of sMg levels is shown in Figure 2. The mean sMg level was 2.12 ± 0.22 mg/dl (median, 2.1 mg/dl; range, 2.0–2.28 mg/dl).

The baseline characteristics of HFpEF patients are shown in Table 1. Overall, patients had a mean age of 71.7 ± 9.4years and 54.6% were male. The mean systolic and diastolic blood pressures were 130.2 ± 20.7 and 71.0 ± 12.6 mm Hg, respectively. The lower sMg group (sMg < 2.0 mg/dl) showed significantly higher prevalence of DM, uric acid and BNP levels compared with the higher sMg group (sMg ≥ 2.0 mg/dl). No significant differences were observed regarding the use of all medications (loop diuretics, mineral-corticoid receptor antagonists, RAS inhibitors, calcium channel blockers, β-blockers, statins, and Mg preparations).

**Table 1:**
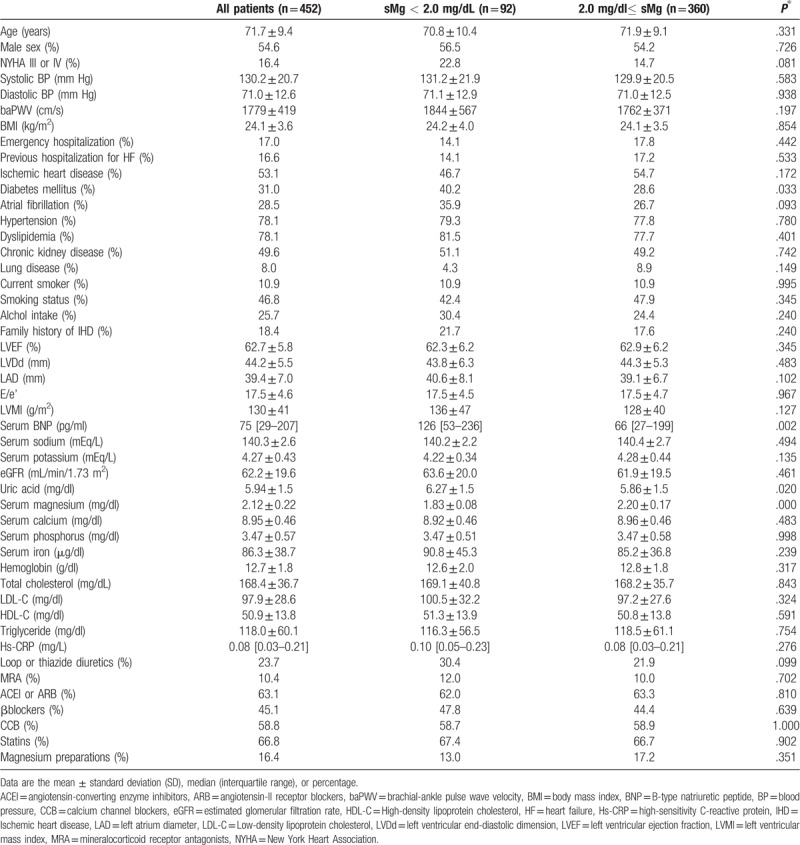
Baseline characteristics of heart failure with preserved ejection fraction (HFpEF) patients according to groups defined by serum magnesium (sMg).

### 3.2 Follow-up

Follow-up data on HF-related events were available for 452 HFpEF patients. The follow-up period was 0 to 50 months (median, 47.3 months) and 48 HF-related events (10.6%) were recorded. No patients were lost to follow-up. The frequency of HF-related events was significantly higher in the lower sMg group compared with the higher sMg group (n = 16, 17.4% vs n = 32, 8.9%; *P* = .018).

### 3.3 Kaplan–Meier curve

**Figure 3:**
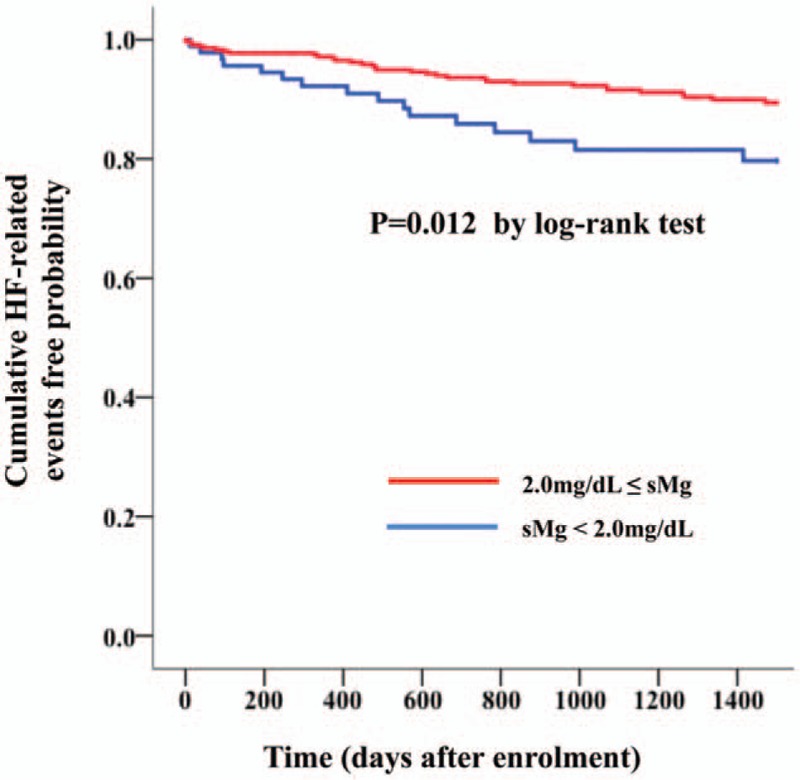
Kaplan–Meier analysis for the probability of heart failure (HF) related events in HF patients with preserved left ventricular ejection fraction according to serum magnesium (sMg) levels. The 0 time point in the x-axis indicates discharge day of the qualifying HF hospitalization.

On the Kaplan–Meier curve, shown in Figure 3, a significantly higher probability of HF-related events was noted in the lower sMg group compared with the higher sMg group (log-rank test, *P* = .012).

### 3.4 Cox proportional hazard model analysis

The results of simple and multivariate regression Cox proportional hazard analysis for HF-related events are shown in Table 2. By univariate Cox hazard analysis, thirteen variables were identified as significant predictors (previous hospitalization for HF, ischemic heart disease, atrial fibrillation, hypertension, chronic kidney disease, LVEF, left atrium diameter, E/e’, LV mass index, serum sodium, hemoglobin, Ln-BNP, and sMg < 2.0 mg/dl). In a multivariate Cox proportional hazard analysis including factors identified in the subanalysis of the I-PRESERVE trial (age, previous hospitalization for HF, DM, and Ln-BNP; 4 prognostic factors [PF4])^[20]^ by forced entry methods, an sMg < 2.0 mg/dl was independently and significantly associated with HF-related events (hazard ratios [HR]: 2.365, 95% confidence intervals [CI]: 1.267–4.413, *P* = .007).

**Table 2:**
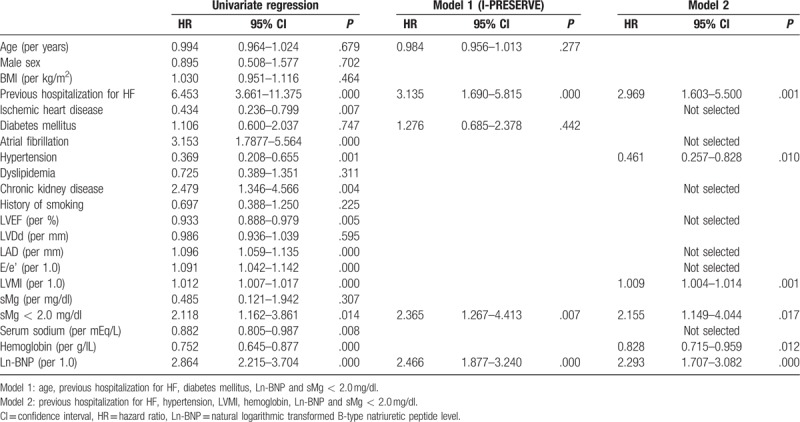
Cox hazard analyses of HF-related events in HFpEF patients.

### 3.5 Continuous net reclassification improvement

We reclassified the risk of HF-related events after adding the lower sMg to the PF4 determined by subanalysis of the I-PRESERVE trial^[20]^; the continuous net reclassification improvement (NRI) was 29.0% (*P* = .041) (Table 3).

**Table 3:**
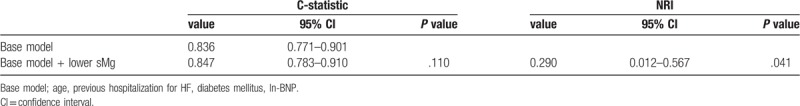
C-Statistics and net reclassification improvemen (NRI) for the Cox hazard model to predict HF-related events in patients with HFpEF by the addition of serum magnesium (sMg) to the base model.

## 4 Discussion

The main feature of the present study was that there was an association between sMg, and HF-related events among a prospective cohort of patients with HFpEF. The main findings were as follows:

1. Kaplan–Meier curve revealed a significantly higher probability of HF-related events in the lower sMg group compared with the higher sMg group;
2. Multivariate Cox-proportional-hazard analysis revealed that the lower sMg group had significantly and independently higher probabilities of HF-related events compared with the higher sMg group;
3. The NRI was significant when the lower sMg level was added to the PF4 (age, previous hospitalization for HF, DM, ln-BNP).

To the best of our knowledge, this report is the first to describe a close association between sMg and HF-related events in patients with HFpEF despite some limitations.

Mg deficiency tends to occur in HF patients. The mechanism of this deficiency is a combination of the following:

1. reduction in calorie intake by anorexia due to gastrointestinal congestion or an absorption disorder from the intestinal tract;
2. Mg^2+^ chelating action enhancement of sympathetic nerve activity and an increase in serum free fatty acid;
3. increases in urinary Mg^2+^ excretion due to secondary aldosteronism and increases in the antidiuretic hormone;
4. increases in urinary Mg^2+^ excretion by diuretics and digitalis used for the treatment of HF; and
5. further aldosterone secretion from the adrenal cortex spherical layer due to hypomagnesemia, which forms a vicious circle.

Secretion of aldosterone is stimulated by K^+^, Ca^2+^, and suppressed by Na^+^, Mg^2+^.

In elderly patients with HF, HFpEF is the more common type compared with non-elderly HF patients.^[21]^ It is believed that, due to aging, the LV experiences more hypertrophy, leading to an increased prevalence of hypertension and that due to aging, LV remodeling, and myocardial fibrosis progress, resulting in a decrease in LV compliance.^[22]^ As the LV diastolic capacity declines, the left atrial pressure rises and the left atrium is expanded, which is a risk factor for future cardiovascular disease.^[23]^ In HFpEF, there are many comorbidities outside the heart, but because elderly people already have various comorbidities related to the body fluid volume and maximum oxygen uptake, such as chronic kidney disease and pulmonary diseases, HF tends to occur, even with mild contraction reduction.^[24]^

Many body fluid electrolyte abnormalities are often found in HF,^[25]^ which is 1 reason why mergers of renal dysfunction are very frequent in HF. Indeed, the results of the present study also showed a significant reduction in renal function in the group with the worst prognosis. In addition, hemodynamic abnormalities (circulatory plasma volume, cardiac function, blood pressure), neurological factors (such as sympathetic nervous system), hormonal actions (such as RAS and vasopressin),^[25]^ and treatments (especially diuretics) are complicatedly involved in the pathophysiology of HF, which is itself a cause of electrolyte abnormality.

No drugs that improve the prognosis of HFpEF patients have been found to date. The Japanese Diastolic Heart Failure (J-DHF) study^[25]^ suggested the usefulness of β-blockers, but in randomized large-scale clinical trials, such as the Perindopril in Elderly People with Chronic Heart Failure (PEP-CHF) trial,^[26]^ which used angiotensin converting enzyme inhibitors, in addition to the Candesartan in Heart Failure-Assessment of Reduction in Mortality and Morbidity-Preserved (CHARM-Preserved) trial^[27]^ and I-PRESERVE trial,^[28]^ both of which used angiotensin receptor antagonists (ARBs), did not show the usefulness of drug treatments. Additionally, the usefulness of isosorbide mononitrate in HF treatment was not found in the Nitrate's Effect on Activity Tolerance in Heart Failure with Preserved Ejection Fraction (NEAT-HFpEF) trial.^[29]^ On the other hand, in the Prospective comparison of ARNi with ARB on Management of heart failure with preserved ejection fraction (PARAMOUNT) trial^[30]^ on HFpEF patients with angiotensin receptor-neprilysin inhibitors (ARNi),^[31,32]^ LCZ696 (valsartan/sacubitril, Entresto) not only improved the NYHA cardiac function classification after 36 weeks compared with HFpEF patients taking an ARB but also improved the renal function. From this observation, ARNi modications are expected to improve the prognosis of HFpEF patients. Spironolactone (Aldactone), an aldosterone antagonist, is recommended in the HF guidelines because it improves the prognosis of HFrEF patients.^[33]^ In the Aldo-DHF trial,^[24]^ spironolactone improved the LV dilations and the N-terminal pro-BNP levels in patients with HFpEF and it significantly decreased HF hospitalizations in the Treatment of Preserved Cardiac Function Heart Failure with an Aldosterone Antagonist (TOPCAT) study.^[34]^ One of the physiological conditions of HFpEF is that due to a sharp increase in left atrial pressure during exercise, pulmonary edema occur can occur. Focusing on the increase in the left atrial pressure, the REDUCe Elevated Left Atrial Pressure in Patients with Heart Failure (REDUCE LAP-HF) study^[35]^ was conducted by mechanically creating a left-to-right shunt with a catheter between the left and right atria to relief pressure and improve symptoms.

Accumulating clinical evidences indicates that hypomagnesemia and hypermagnesemia are associated with a worse outcome in HF, according to a meta-analysis that was performed recently.^[36]^ However, these cited references included a combination of HFrEF and HFpEF patients, and no examination limited to HFpEF patients has previously been performed. These 2 pathological conditions are largely different, and as mentioned above, their therapeutic effects, as well as the prognosis,^[9,10]^ are different; thus, we believe that HFrEF and HFpEF patients should be managed differently.

Because we did not investigate the biological basis for the determination of the HF-related event rates according to sMg that we found, little is known about the potential underlying mechanisms. However, we expect further examinations will be performed on this topic, including animal experiments.

Our findings indicate that lower sMg levels significantly correlate with HF-related events in patients with HFpEF. After adjusting for various clinical parameters, a low sMg level is still an independent predictor. The NRI was significant when the lower sMg level was added to the PF4. The underlying mechanisms of HF-related events in the low Mg group in HFpEF patients still remain unknown. Although we mentioned above that HFpEF and HFrEF differed according to pathological conditions, we believed that results similar to those found in HFrEF patients^[37–39]^ will depend on HF treatments and the nutritional statuses caused by the treatments, rather than the mechanism of HF.

In the present study, we mentioned the importance of sMg management in HFpEF. There is a possibility for improving the prognosis of cardiovascular disease itself by positively intervening against Mg concentration abnormalities in the future. Therefore, our present work provides data that indicates that sMg levels not only provide important prognostic information regarding HFpEF patients but also that targeting optimal sMg levels might be a promising therapeutic target of HFpEF. Furthermore, recently, new classification criteria for HF have been proposed, and it is desirable to identify the pathology of each disease condition.

### 4.1 Study limitations

The present study has some limitations. First, it was a single center design with a relatively small population. Therefore, a larger multiracial and multicenter study is required. Second, there were fewer patients with sMg < 2.0 mEq/L compared with the other group, which is thought to be because the patient samples were collected after medical therapy was initiated. Third, the group with the worst prognosis had significantly worse renal function. Thus, there is the possibility that a poor prognosis associated with a decline in renal function, rather than an electrolyte, could not be ruled out.

### 4.2 Future directions

Together with the aging society, which is progressing rapidly worldwide, cases of HF are steadily increasing, and 500,000 individuals are newly diagnosed in the United States annually as HF,^[40]^ which is also a social problem. Under these circumstances, understanding of pathology of HFpEF and appropriate interventions are considered important subjects in the future. Although the pathology of HFpEF remains poorly understood, the establishment of a new risk stratification tool is an urgent issue in modern society. The specific factors influencing the association between sMg and HF are unclear, and the extent to which these factors may contribute to the relationships of both sMg and the promotion of HF risks is unknown. Thus, further pathophysiological and molecular physiological studies, including animal experiments, are warranted. Although the sMg levels is highly expected to have clinical value, large-scale clinical studies are required to confirm its value. Therefore, additional detailed, prospective, multi-center studies are warranted to verify this precise usefulness.

## 5 Conclusions

Despite the limitations of our study, we demonstrated that lower sMg level was significantly correlated with the occurrence of future HF-related events in HFpEF patients. sMg level might be able to successfully predict future HF-related events, and management of sMg in HFpEF patients might be thus important.

## Acknowledgments

We thank all of the paramedical staff and clinical secretaries for their kind support during this work.

## Author contributions

**Conceptualization:** Daisuke Sueta, Koichiro Fujisue.

**Data curation:** Taiki Nishihara, Daisuke Sueta.

**Formal analysis:** Taiki Nishihara, Fumi Oike.

**Funding acquisition:** Daisuke Sueta.

**Investigation:** Taiki Nishihara, Daisuke Sueta, Koichiro Fujisue, Hiroki Usuku, Masafumi Takae,

**Methodology:** Taiki Nishihara, Daisuke Sueta.

**Project administration:** Eiichiro Yamamoto, Kenichi Tsujita.

**Resources:** Taishi Nakamura, Hiroaki Kawano.

**Supervision:** Hirofumi Soejima, Koichi Kaikita.

**Validation:** Seiji Takashio, Yuichiro Arima, Satoshi Araki

**Visualization:** Satoru Suzuki, Kenji Sakamoto.

**Writing – original draft:** Taiki Nishihara, Daisuke Sueta.

**Writing – review & editing:** Eiichiro Yamamoto.

## Supplementary Material

Supplemental Digital Content
