# Supplementary material for: Clinical significance of serum magnesium levels in patients with heart failure with preserved ejection fraction
Source: Medicine (Baltimore). 2019 Sep 20;98(38):e17069. doi: 10.1097/MD.0000000000017069 (PMC6756625; doi:10.1097/MD.0000000000017069)
Supplement: Supplemental Digital Content [file medi-98-e17069-s001.docx]

Supplemental Content

**Clinical Significance of Serum Magnesium Levels in Patients with Heart Failure with Preserved Ejection Fraction**

Taiki Nishihara, MD, Eiichiro Yamamoto, MD, PhD, Daisuke Sueta, MD, PhD, Koichiro Fujisue, MD, PhD, Hiroki Usuku, MD, PhD, Fumi Oike MD, Masafumi Takae, MD, Yuichiro Arima, MD, PhD, Satoshi Araki, MD, PhD, Seiji Takashio MD, PhD, Taishi Nakamura, MD, PhD, Satoru Suzuki, MD, PhD, Kenji Sakamoto, MD, PhD, Hirofumi Soejima, MD, PhD, Hiroaki Kawano, MD, PhD, Koichi Kaikita, MD, PhD, Kenichi Tsujita, MD, PhD

**Affiliation:** Department of Cardiovascular Medicine, Faculty of Life Sciences, Graduate School of Medical Science and Center for Metabolic Regulation of Healthy Aging (CMHA), Kumamoto University, Kumamoto, Japan

Supplemental Content

***Ethics statement***

All procedures were conducted in accordance with the Declaration of Helsinki and its amendments. The study protocol was approved by the institutional review board of Kumamoto University (approval number, Senshin 2225). This study is registered at the University Hospital Medical Information Network (UMIN) Clinical Trials Registry (UMIN000036884). Opt-out materials are available at: http://www.kumadai-junnai.com/home/wp-content/uploads/houkatsu.pdf.

***Clinical parameters***

The clinical parameters were described previously.[1, 2, 3, 4] In brief, the baseline demographic data, cardiovascular risk factors, and medications on discharge were documented. Hypertension was defined as a recorded blood pressure >140/90 mmHg or taking any antihypertensive medications as described previously.[1, 4] Diabetes mellitus (DM) was defined as the presence of symptoms of diabetes and a random plasma glucose concentration ≥200 mg/dL; fasting plasma glucose concentration ≥126 mg/dL; and 2-hr plasma glucose concentration ≥200 mg/dL according to an 75 g oral glucose tolerance test or taking any medications for DM. Dyslipidemia was defined as low-density lipoprotein levels ≥140 mg/dL (≥3.63 mmol/L), high-density lipoprotein levels <40 mg/dL (1.04 mmol/L) or triglycerides ≥150 mg/dL (≥1.7 mmol/L) or taking any medications for dyslipidemia. Current smoking status was determined via an interview.

***Echocardiography***

Echocardiography was performed under stable conditions on admission by experienced cardiac sonographers who had no knowledge of the study data. Left ventricular ejection fraction (LVEF) was measured using a modified Simpson’s method. The LVEF; ratio of early transmitral flow velocity to early diastolic mitral annular velocity (E/e’), which was assessed by tissue Doppler; and LV mass index (LVMI) were measured by echocardiography (Vivid 7®; GE-Vingmed Ultrasound, Horton, Norway; Aplio XG®; Toshiba, Tokyo, Japan) as previously reported. [1, 5]

***Biochemistry***

Patient`s B-type natriuretic peptide (BNP) levels were analyzed using a commercially available assay (Abbott Japan, Matsudo, Japan) in the hospital clinical laboratory on admission. BNP levels were transformed into natural logarithmic levels (ln-BNP) for a normal distribution. The estimated glomerular filtration rate was calculated using the Japanese Society of Nephrology formula.[6]

***Follow-up and HF-related events***

Patients were followed up at our outpatient clinics until October 2014 or until the occurrence of hospitalization for HF decompensation. We defined hospitalization for HF decompensation as hospital admittance due to HF-related events. HF-related events were ascertained from a review of medical records and confirmed by direct contact with patients, their families, their physicians, or by an annual telephone interview conducted with each patient. Hospitalization for HF decompensation was diagnosed if the patient was admitted with symptoms typical of HF and had objective signs of worsening HF that required intravenous drug administration.

***Statistical analysis***

The software Statistical Package for Social Science(SPSS) ver. 22.0 (IBM Japan, Tokyo, Japan) was used for statistical analyses. Non-normally distributed data are expressed as the median (interquartile range), and P-values <0.05 were considered statistically significant. Differences between two groups were assessed by the chi-squared test for categorical variables, and Student’s unpaired t-test or the Mann–Whitney U-test (as appropriate) were used for difference between for continuous variables. We defined lower sMg as < 2.0 mg/dL(=0.8 mmol/L) based on recent review concerning the relationship between sMg levels and cardiovascular events.[7] In the present study, the association between sMg and the HF-related events rate was investigated using Kaplan-Meier estimates (serum Mg cut-off, 2.0 mg/dL). A Kaplan–Meier curve was used to determine the cumulative incidence of HF-related events, and the log-rank test was used to compare the incidence of HF-related events between groups. Using the univariate Cox proportional hazards model, we evaluated the association between sMg and HF-related events. We constructed the following combination of significant factors in the univariate Cox analysis to avoid the over-fit problem with limited outcomes in the present study: We tested the multivariate Cox hazard analyses by the forced inclusion model (Model 1) and the stepwise model (Model 2). Model 1; age, previous hospitalization for HF, DM, ln-BNP (four prognostic factors; PF4) determined via subanalysis of the Irbesartan in Heart Failure with Preserved Ejection Fraction Study (I-PRESERVE) trial[8] and sMg < 2.0 mg/dL (adjusted for PF4). These were the most powerful factors associated with cardiovascular events. Model 2; previous hospitalization for HF, atrial fibrillation (AF), hypertension (HT), LVMI, hemoglobin, Ln-BNP and serum Mg < 2.0 mg/dL; as clinically relevant variables that were significant factors in the univariate Cox analysis (p<0.01). Furthermore, the estimates of C-statistics in the Cox proportional hazards regression models were compared after the addition of lower sMg to the PF4. We also assessed the incremental effects of adding lower sMg levels to the PF4 to predict HF-related events using the net reclassification index (NRI).

**References**

1 Tabata N, Sueta D, Yamamoto E, et al. Outcome of current and history of cancer on the risk of cardiovascular events following percutaneous coronary intervention: a Kumamoto University Malignancy and Atherosclerosis (KUMA) study. *Eur Heart J Qual Care Clin Outcomes* 2018;**4**:290-300.

2 Fujisue K, Tokitsu T, Yamamoto E, et al. Prognostic significance of polyvascular disease in heart failure with preserved left ventricular ejection fraction. *Medicine (Baltimore)* 2019;**98**:e15959.

3 Sueta D, Yamamoto E, Nishihara T, et al. H_2_FPEF Score as a Prognostic Value in HFpEF patients. *Am J Hypertens* 2019. doi: 10.1093/ajh/hpz108.

4 Tabata N, Sueta D, Yamamoto E, et al. A retrospective study of arterial stiffness and subsequent clinical outcomes in cancer patients undergoing percutaneous coronary intervention. *J Hypertens* 2019;**37**:754-64.

5 Nishihara T, Tokitsu T, Sueta D, et al. Serum Potassium and Cardiovascular Events in Heart Failure With Preserved Left Ventricular Ejection Fraction Patients. *Am J Hypertens* 2018;**31**:1098-105.

6 Matsuo S, Imai E, Horio M, et al. Revised equations for estimated GFR from serum creatinine in Japan. *American Journal of Kidney Diseases* 2009;**53**:982-92.

7 Ter Braake AD, Shanahan CM, de Baaij JHF. Magnesium Counteracts Vascular Calcification: Passive Interference or Active Modulation? *Arterioscler Thromb Vasc Biol* 2017;**37**:1431-45.

8 Anand IS, Rector TS, Cleland JG, et al. Prognostic value of baseline plasma amino-terminal pro-brain natriuretic peptide and its interactions with irbesartan treatment effects in patients with heart failure and preserved ejection fraction: findings from the I-PRESERVE trial. *Circulation: Heart Failure*;4(5):569-77.
